# Process description and evaluation of Canadian Physical Activity Guidelines development

**DOI:** 10.1186/1479-5868-7-42

**Published:** 2010-05-11

**Authors:** Mark S Tremblay, Michelle E Kho, Andrea C Tricco, Mary Duggan

**Affiliations:** 1 Healthy Active Living and Obesity Research Group (HALO), Children's Hospital of Eastern Ontario Research Institute, 401 Smyth Road, Ottawa, ON, K1H 8L1 Canada; 2 Department of Clinical Epidemiology and Biostatistics, McMaster University, 1200 Main Street West, MDCL 3200, Hamilton, ON, L8N 3Z5 Canada; 3 Population Health Program, University of Ottawa, 1 Stewart Street, Ottawa, ON, K1N 6N5 Canada; 4 Canadian Society for Exercise Physiology, 185 Somerset Street West, Suite 202, Ottawa, ON, K2P 0J2 Canada

## Abstract

**Background:**

This paper describes the process used to arrive at recommended physical activity guidelines for Canadian school-aged children and youth (5-17 years), adults (18-64 years) and older adults (≥65 years).

**Methods:**

The Canadian Society for Exercise Physiology (CSEP) Physical Activity Measurement and Guidelines (PAMG) Steering Committee used the Appraisal of Guidelines for Research Evaluation (AGREE II) Instrument to inform the guideline development process. Fourteen background papers and five systematic reviews were completed. Systematic review authors appraised and synthesized the data, and proposed specific recommendations at an international consensus conference of invited experts and key stakeholders. Independently, an international panel of experts interpreted the evidence from the systematic reviews and developed recommendations following attendance at the Consensus Conference.

**Results:**

Using the AGREE II instrument as a guide, specific *foci *for each of the guidelines were defined and systematic review methodology was used to synthesize the evidence base. The expert panel, CSEP PAMG Steering Committee and methodological consultants reviewed the systematic reviews and Consensus Statement. The expert panel achieved consensus on the level of evidence informing the physical activity guidelines and developed a separate document outlining key recommendations, interpretation of the evidence and justification of each recommendation.

**Conclusion:**

The CSEP and Public Health Agency of Canada followed a rigorous process to examine the evidence informing potential revisions to existing physical activity guidelines for Canadians. It is believed that this is the first physical activity guideline development process in the world to be guided and assessed by AGREE II and AMSTAR instruments.

## Background

Over the past several decades habitual physical activity among Canadians has decreased, while corresponding increases in obesity and prevalence of chronic disease have been observed [1]. Engaging in regular physical activity reduces the risk of developing chronic disease and contributes to overall health [1-4]. Since 1995, the Canadian Society for Exercise Physiology (CSEP) and Public Health Agency of Canada - Centre for Health Promotion have collaborated on the development of physical activity guidelines to preserve and promote the health of Canadians, to help Canadians become more aware of the benefits of physical activity, and to encourage Canadians of all ages to become more physically active.

The mandate of CSEP is "to promote the generation, synthesis, transfer and application of knowledge and research related to exercise physiology (encompassing physical activity, fitness, health, nutrition, epidemiology, and human performance)." [5] With a membership of approximately 4000 individuals across Canada, the CSEP actively encourages scientific investigators to pursue new knowledge in all areas of human movement physiology, and to publish and interpret their results. The CSEP's Health and Fitness Program is involved in the application of this knowledge to develop and improve physical activity and health strategies to enhance human health, reduce the prevalence of overweight and obesity, and reduce the prevalence and alleviate the symptoms of acute and chronic diseases or conditions. The CSEP is unique in that it provides access to relevant, high-quality cutting-edge science through their member's research and organizational liaisons, and facilitates translation of this knowledge to health and fitness professionals from many physical activity-related fields for use in their professional practice. The CSEP also ensures that the new knowledge is incorporated into informative, practical educational materials for investigators, health and fitness professionals, and the general public.

The Public Health Agency of Canada is an agency of the Government of Canada that is responsible for public health - the science and art of preventing disease, prolonging life and promoting health through the organized efforts and informed choices of society, organizations, public and private, communities and individuals. Its mission is to promote and protect the health of Canadians through leadership, partnership, innovation and action in public health. Among its responsibilities for public health, the Public Health Agency of Canada has the lead on the Government of Canada's policy regarding physical activity, the objectives of which are to:

(a) promote physical activity as a fundamental element of health and well-being;

(b) encourage all Canadians to improve their health by integrating physical activity into their daily lives; and

(c) assist in reducing barriers faced by Canadians that prevent them from being active.

The Healthy Living Program, part of the federal Healthy Living and Chronic Disease Initiative, is a cornerstone of the Public Health Agency of Canada's health promotion efforts and a proactive response to the rapid increase in chronic disease across population groups. It aims to lead, foster and support action to address the conditions that support physical activity, healthy eating, and healthy weights for all Canadians and with particular emphasis on sub-populations experiencing health disparities. The Program encompasses a range of initiatives, tools and strategies that seek to directly impact key determinants of health; for example by fostering the creation of health-supporting social and physical environments, seeking to optimize personal health practices, and laying the groundwork for healthy child development. The CSEP, in partnership with the Public Health Agency of Canada, led the process to develop each of Canada's Physical Activity Guidelines and Guides [6-15] and convened the Steering Committee guiding the process to review current evidence underlying physical activity measurement and guidelines in Canada.

## Overview of the Project

In 1995 the CSEP and the Public Health Agency of Canada (then Health Canada) began work on the development of physical activity guidelines for apparently healthy adults aged 20-55 years. Canada's Physical Activity Guide to Healthy Active Living was released in 1998 [6]. In 1999, CSEP and the Public Health Agency of Canada released Canada's Physical Activity Guide for older adults (aged 55+ years) [7], and in 2002, Canada's Physical Activity Guides for children (aged 6-9 years) [8-11] and youth (aged 10-14 years) [12-15]. Canada's Physical Activity Guides are the Public Health Agency of Canada's most requested resources. Details of the development and assessment of the family of guidelines and guides are available elsewhere [16-18].

The purpose of this manuscript is to describe and report on the process used to inform potential revisions to Canadian Physical Activity Guidelines. The process details of the Physical Activity Measurement and Guidelines project (PAMG project) are described, the relationship of this project with other domestic and international initiatives is discussed, and the quality of the guideline development process is reported using the Appraisal of Guidelines, Research, and Evaluation (AGREE II) Instrument [19,20].

## Methods

### History of the current process

**Figure 1:**
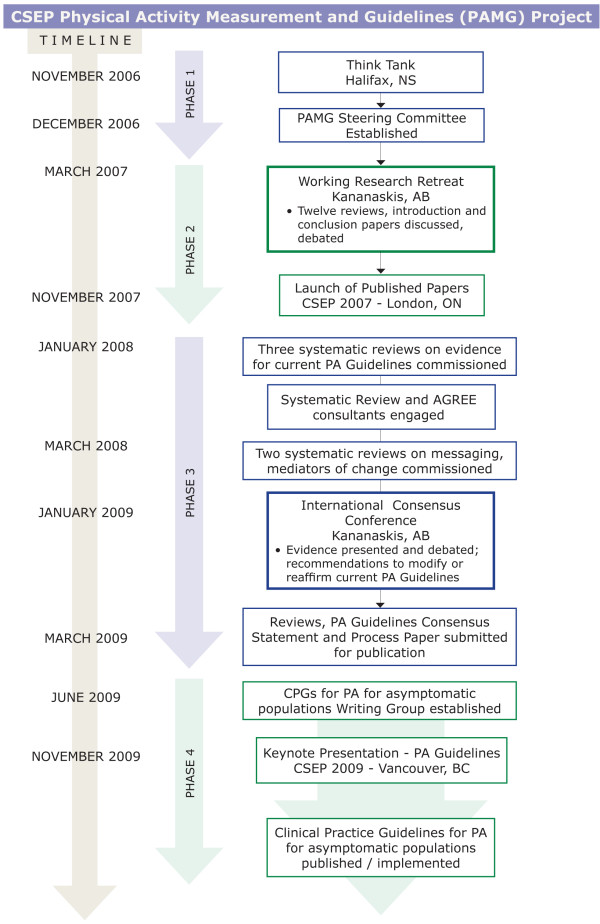
**This figure outlines the history of the guidelines development process from November 2006 to present**.

To arrive at a Canadian consensus on appropriate physical activity guidelines for school-age children, adults and older adults, we undertook the following steps (Figure 1):

Phase 1 (November 2006 - March 2007)

• A Think Tank meeting of physical activity experts (including many who had developed the existing Canadian Physical Activity Guides) was held in Halifax in November 2006. The primary objectives of the meeting were to:

◦ discuss the nature of physical activity guidelines, prescriptions and recommendations;

◦ discuss the messaging, delivery, expectations and effectiveness of physical activity guides;

◦ share current findings on physical activity monitoring and surveillance as they relate to the assessment of people meeting physical activity guidelines;

◦ provoke detailed discussions on the current state of understanding in this area, including potential benefits or limitations of existing physical activity guidelines;

◦ discuss whether certain groups need special guidelines; and,

◦ initiate a review of Canada's Physical Activity Guidelines and Guides.

• Following this meeting, the Public Health Agency of Canada invited the CSEP to submit a proposal to move forward on the recommendation to commission a series of background papers that would address the questions raised in the Think Tank.

• The CSEP convened a Steering Committee on the PAMG Project with representatives from the CSEP and the Public Health Agency of Canada (Appendix A). This Committee met in December 2006 to identify the evidence-informed review topics, the paper authors who would be approached and to set a date for a Working Research Retreat where these papers would be presented. Twelve papers were commissioned: six papers addressing physical activity guidelines for population-specific age groups (children and youth [2], adults [1], older adults [3], pre-school children [21]) and specific populations (Aboriginal Canadians [22], Canadians with a disability [23]) and six others that addressed issues surrounding the history of physical activity guidelines development in Canada [17]; effective messaging [24]; impact of the current Physical Activity Guides [16]; physical activity measurement limitations [25]; physical activity profiling and measurement advances [26]; and the role of incidental physical activity and sleep on physical activity - health relationships [27]. It is important to note that the Steering Committee made a conscious decision to try to seek authors who were acknowledged experts in their area but also up-and-coming in their academic career rather than only the investigators who had been involved in the earlier Guides' development. An introductory [18] and concluding paper [28] were also prepared to contextualize, explain and summarize the commissioned papers.

Phase 2 (March 2007 - December 2007)

• In March 2007, the authors met to review the evidence presented in the commissioned papers, discuss and debate the findings and reach consensus on what the evidence indicated. The twelve papers, and an introduction and conclusion, were subsequently revised based on the discussion and following review by three external referees, and published in a special joint supplement of the *Canadian Journal of Public Health/Applied Physiology, Nutrition and Metabolism *in November 2007 [1-3,16-18,21-29])

• Findings from the papers were presented by the authors and Steering Committee members at numerous conferences commencing with the CSEP Annual Meeting in London, Ontario in November 2007.

• In December 2007 the CSEP submitted a 'vision' document entitled "Future Directions: Physical Activity Measurement and Guidelines in Canada" to the Public Health Agency of Canada that outlined a multi-year plan for updating the current physical activity guidelines, including the development of Clinical Practice Guidelines (CPGs) for physical activity for asymptomatic individuals and developing guidelines for population segments not currently represented (e.g., preschool, teens aged 15-19 years). The proposal also addressed the need to be proactive and innovative in order to optimize the use of emerging technologies to more effectively convey physical activity guidelines through personalization and interactivity.

Phase 3 (January 2008 - March 2009)

• The CSEP and the Public Health Agency of Canada elected to move forward with developing CPGs for physical activity. The lead authors of the three relevant papers in the 2007 Supplement (Janssen [2]; Warburton [1]; Paterson [3]) were commissioned to perform systematic reviews on their respective population segments (children and youth, adults and older adults) [30-32]. To extend comprehensiveness, it was agreed that two additional reviews would be commissioned to address modifiable mediators of physical activity behaviour change [33] and effective physical activity messaging [34] evidence for healthy adults.

## Concurrent processes

Three important concurrent processes occurred during CSEP's development of the physical activity guidelines that provoked increased attention to guideline development quality, these include: harmonization with other Canadian cardiovascular guideline initiatives; recruitment of methodological consultants; and informal collaborations with international physical activity guideline development groups. These concurrent processes influenced when and how we did things, and they influenced who we involved in our processes.

### Harmonization with other Canadian guideline initiatives

In June 2007, the Canadian Vascular Coalition approached CSEP about producing physical activity CPGs similar to those for hypertension [35,36] and obesity [37,38], to enhance acceptability by the medical community [39]. The Canadian Vascular Coalition is an informal group of health professionals (Appendix B) with a significant interest in the development, dissemination, evaluation, integration and harmonization of evidence-based CPGs specifically aimed at improving the vascular health of Canadians; CSEP is a member of the Canadian Vascular Coalition representing 'physical inactivity' as a risk factor for vascular disease. The Canadian Vascular Coalition's goal is to integrate and harmonize the production of Canadian CPGs in all aspects of vascular health, particularly in physical activity and smoking cessation. Although the weight of scientific evidence regarding the benefits of these two interventions in vascular health is overwhelming, they currently lacked a CPG structure similar to the other vascular risk groups in the Canadian Vascular Coalition. Obesity Canada is also a member of the Canadian Vascular Coalition and CSEP agreed that a uniform approach would help achieve the Canadian Vascular Coalition's goal to harmonize how CPGs for vascular health are developed and updated. Accordingly, the CSEP adopted steps to ensure rigour, comprehensiveness and transparency in the CPG process, adopted the AGREE CPG development protocol [19,20,40] and used the same evidence assessment system as the CPGs for the Prevention and Treatment of Obesity in Children and Adults [38].

Recently, in Canada there has been substantial interest and effort in establishing processes and mechanisms to harmonize, standardize and connect efforts related to health promotion and disease prevention CPG development and implementation. In addition to the efforts of the Canadian Vascular Coalition mentioned above, the C-CHANGE (Canadian - Cardiovascular HArmonized National Guidelines Endeavour) project for risk intervention has been initiated by the Canadian Institutes of Health Research, Institute of Circulatory and Respiratory Health in collaboration with the Canadian Vascular Coalition and the Canadian Heart Health Strategy. The goal of C-CHANGE is to produce a simple harmonized set of state-of-the-art guidelines to serve the broad primary care medical community in managing cardiovascular disease risk factors, including physical inactivity.

The Public Health Agency of Canada has very recently reconstituted and revitalized its Canadian Prevention Task Force on Preventive Health Care (see http://www.phac-aspc.gc.ca/cd-mc/ctfphc-gecssp-eng.php). "*The Task Force will address the need for streamlined access to credible, up-to-date and relevant evidence to support primary care practice... it will work with a variety of health professional groups and non-governmental organizations to support development of prevention tools and activities to aid implementation of clinical guidelines in practice*."

In April 2009 the Canadian Partnership Against Cancer http://www.partnershipagainstcancer.ca/index.html launched its Coalitions Linking Action and Science for Prevention (CLASP) initiative. The goal of CLASP is to integrate science, practice and policy, to inform and influence comprehensive chronic disease prevention programs and policy initiatives and investments. The Canadian Partnership Against Cancer, through CLASP will fund coalitions in Canada to pursue this goal.

The Canadian Vascular Coalition, C-CHANGE, Canadian Prevention Task Force on Preventive Health Care, and CLASP initiatives provide overwhelming evidence of a movement in Canada towards harmonizing, standardizing and connecting efforts to develop and implement robust, evidence-informed guidelines and practices for the prevention of chronic disease. The PAMG project is the physical activity scientific community's response to this movement.

### Recruitment of methodological consultants

In March 2008, the CSEP engaged two methodological consultants to advise the PAMG Steering Committee regarding best practices for conducting the systematic reviews of evidence and developing physical activity guidelines in a fashion suitable for the development of CPGs. While Canadian experts in physical activity research conducted the systematic reviews, few had any previous systematic review experience. To ensure that the systematic review process was consistent across reviews and was of high methodological rigour, CSEP consulted a systematic review expert (ACT) with experience with close to 20 systematic reviews or systematic review methodology projects. The systematic review consultant provided documents outlining the review process, and assistance to the review authors whenever necessary. The practice guideline consultant (MEK), a physiotherapist with graduate training in health research methodology, was responsible for documenting and advising on the guideline development process.

Upon completion of the systematic reviews, the systematic review expert consultant and the guideline consultant critically appraised the reviews and provided feedback to the review authors. Both expert consultants focused on the methodological rigour of the review process itself versus the scientific content of the papers. The authors incorporated the revisions and presented the revised papers at an International Consensus Conference on physical activity guidelines in January 2009. The review authors incorporated further comments and suggestions from the conference into the systematic reviews.

### Informal collaborations with international physical activity guideline development groups

In January 2007, CSEP initiated efforts to consult on and potentially harmonize physical activity guidelines with developers from other countries and international organizations (i.e., United Kingdom, United States, World Health Organization, Australia).

In April 2007, the British Association of Sport and Exercise Sciences (BASES) convened a Consensus Meeting on Physical Activity in the Prevention of Chronic Disease, with the experts in obesity, type 2 diabetes, cardiovascular disease, cancer and psychological wellbeing invited to produce the BASES consensus statement on physical activity in the prevention of chronic disease. The expert panel focused on prevention rather than treatment. The panel considered minimal and optimal levels of physical activity, physical activity in children and adolescents, the prevention of exercise-induced injury, and population-specific exercise guidelines. The PAMG Project sent a delegate to the BASES meeting to observe and participate. Unfortunately the BASES process has thus far not produced new population specific physical activity or exercise guidelines.

The United States Physical Activity Guidelines Advisory Committee (PAGAC) invited PAMG representatives to attend a meeting in the United States in late February 2008, where the PAGAC presented and debated their recommendations based on their extensive review and interpretation of the literature compiled by the United States Centers for Disease Control and Prevention. In reciprocation, the Steering Committee invited members of the PAGAC to Canada to meet in March 2008 to discuss the respective initiatives and explore potential avenues of collaboration.

In April 2008 the PAMG Project initiated and participated in a featured symposium at the *International Congress on Physical Activity and Public Health *held in Amsterdam on the 'Development of National Guidelines for Physical Activity and Health - Experiences from Canada and the United States'. The symposium presented to the international audience the process and cross-talk between the two parallel initiatives.

The World Health Organization, under the leadership of Dr. Tim Armstrong, is simultaneously developing global physical activity guidelines. Experts from the PAMG Project have been and continue to be closely linked in to this project.

In addition to these specific events, regular contact between the PAMG Project and the United States PAGAC occurred, including several informal meetings. Furthermore, several representatives from the United States PAGAC, the United Kingdom, Australia and the World Health Organization participated in the International Consensus Conference in January 2009. Increased international and cross-sectoral cooperation of this nature can reduce duplication of effort, accelerate guideline revisions, improve the quality of guideline development processes, increase harmonization of findings and messaging, and improve scientific rigor and should be encouraged.

## Guideline development process

We sought a methodologically rigorous and transparent approach to guideline development. Using the AGREE II instrument as a guide [19,20], we defined specific foci for each of the guidelines and used systematic review methodology to synthesize our evidence base [41]. In this section, we describe our overall methodological approach to guideline development, and highlight how each of the guidelines addressed internationally endorsed standards for guideline reporting.

The AGREE II instrument is a 23-item tool representing 6 quality domains that outline key reporting criteria for practice guidelines [19,20]. Briefly, the original AGREE instrument was developed by an international collaboration of guideline developers through rigorous psychometric methods, including item generation, item reduction, and factor analysis [40]. The purpose of the AGREE instrument is threefold: guideline assessment, guidance for guideline reporting, and guidance for guideline development. AGREE II is a methodological refinement of AGREE, following feedback from key CPG stakeholders [19,20]. In this section, we outline how we used the AGREE, and more recently, the AGREE II instrument to inform our guideline development process and direct readers to specific parts of each guideline for further detailed information. In some cases, the information reported herein applies to all of the guidelines in this collection of papers; in other instances, we will specifically indicate where readers may find information in the papers. In the next sections, we briefly describe each of the six AGREE domains and address how we used the AGREE II instrument to inform our process for each domain.

Guideline process reporting by AGREE domain:

The *Scope and Purpose *domain (3 items) reports the overall guideline aim, specific healthcare questions and target population. Table 1 outlines each of these characteristics by guideline.

**Table 1:** Overview of AGREE Scope and Purpose Items for the physical activity guidelines

Guideline	Children and Youth	Adults	Older Adults
1. The overall objective(s) of the guideline is (are) specifically described	The purpose of this guideline is to provide the rationale for population-based physical activity guidelines in healthy children between 5 and 17 years old to prevent or improve the following 7 health measures: high cholesterol, high blood pressure, metabolic syndrome, obesity, low bone density, depression, and injuries.	The purpose of this guideline is to provide the rationale for population-based physical activity guidelines in healthy adults between 18 and 64 years old on all-cause mortality and to prevent or improve the following 7 chronic health conditions: cardiovascular disease (except stroke), stroke, hypertension, colon cancer, breast cancer, type 2 diabetes, and osteoporosis.	The purpose of this guideline is to provide the rationale for intensity and volume of aerobic physical activities, and for the adjunct of resistance (strength) training for healthy adults ≥65 years of age on functional outcomes including physical limitations, disability, and cognitive losses.
2. The clinical question(s) covered by the guideline is (are) specifically described	How much physical activity is needed for minimal and optimal health benefits?	Is there a dose-response relationship between:	What is the relationship of physical activity with functional outcomes -- to prevent limitations, disability, cognitive losses?
	What types of activity are needed?	Physical activity and all-cause mortality.	
	Does intensity matter?		
	Do the effects vary by sex or age?	Physical activity and the incidence of the following 7 chronic conditions: cardiovascular disease (except stroke), stroke, hypertension, colon cancer, breast cancer, type 2 diabetes, and osteoporosis.	What is the influence of exercise training programs influence on functional outcomes?
3. The patients to whom the guideline is meant to apply are specifically described	Healthy children and youth, 5-17 years old	Healthy adults, 18--64 years old	Healthy adults ≥65 years old <85 years

The *Stakeholder Involvement *domain (3 items) reports how the guideline reflects the perspectives of the intended users. The guideline development group for all of the guidelines included over 25 people from 7 different countries, and several disciplines, including exercise physiology, behavioural science, end users, and methodologists. A five-member independent multidisciplinary international expert panel developed the guideline recommendations. Physical activity guideline developers from the United States, United Kingdom, Australia, and the World Health Organization (Geneva) shared their experiences from the perspectives of their jurisdictions. End-user representatives from ParticipACTION, the Ontario Ministry of Health Promotion, First Nations and Inuit Health Branch, Health Canada and the Public Health Agency of Canada represented key stakeholders from policy and implementation perspectives. The guideline development group did not include citizens from the general public. The consensus document reflects recommendations for children and youth (and their intermediaries), adults, and older adults, who are the intended users or recipients of the recommendations. Figure 1 provides an overview of the consultation process, and Appendix C lists the guideline development group members, expertise, and role(s).

The *Rigour of Development *domain (8 items) examines how the evidence was assembled and synthesized, how the recommendations were developed, and updating mechanisms for the guidelines. Please see each systematic review for details specific to each research question, including search strategies and evidence selection criteria [30-34]. The authors of each systematic review critically appraised individual studies using previously developed quality assessment tools and then reflected on the body of evidence, considering the scientific validity of the studies and overall quality of the evidence. Systematic review authors assigned an overall quality rating to the body of evidence and developed draft recommendations for further consideration by the Consensus expert panel [38]. The Consensus panel, CSEP PAMG Steering Committee and methodological consultants reviewed the five reviews [30-34]. The five review authors, the Steering Committee and both methodological consultants reviewed the Consensus Statement [42]. After further revisions, the Steering Committee approved the final versions presented herein.

In order to facilitate scoring the rigour of development domain, the methodological quality of the systematic reviews was assessed by the methodological experts using the 'assessment of multiple systematic reviews' (AMSTAR) tool [43]. The AMSTAR tool is an 11-item tool which addresses: 1) whether a systematic review protocol was produced, 2) if study selection and data abstraction was done in duplicate, 3) comprehensiveness of the literature search, 4) if the inclusion criteria involved exclusion based on publication status (i.e., grey or unpublished literature), 5) whether a list of included and excluded studies was reported, 6) whether the included study characteristics were reported, 7) if the scientific quality of included studies was assessed, 8) if the study quality was adequately addressed in the conclusions, 9) if the methods used to combine the data were appropriate, 10) if the likelihood of publication bias was assessed, and 11) whether conflict(s) of interest were reported. The AMSTAR tool has recently been shown to have high inter-rater reliability and construct validity [44].

Overall, the 5 systematic reviews scored well on the AMSTAR tool (see assessment in Table 2). The systematic reviews consistently scored well on describing the planned review design (5/5 reviews), conducting duplicate study selection and data abstraction (4/5), conducting a comprehensive literature search (5/5), adequately reporting the study characteristics of included studies (5/5), appropriately assessing the quality of included studies (5/5), appropriately using the study quality formulate conclusions 5/5), clearly reporting study combining methods (5/5), and reporting potential conflicts of interest (5/5). The systematic reviews would be improved if they included unpublished material (0/5), provided a list of excluded studies (1/5), and assessed publication bias (0/5).

**Table 2:** AMSTAR methodological quality assessment of PAMG systematic reviews

Item	**Janssen **[30]	**Warburton **[31]	**Paterson **[32]	**Rhodes **[33]	**Latimer **[34]
1. Was an "a priori" design provided?	Yes	Yes	Yes	Yes	Yes
2. Was there duplicate study selection and data extraction?	Yes, but no strategy to resolve disagreements	Yes, but no info on how to resolve disagreements re: data extraction. (Screening, data abstraction)	Yes	Yes for search, but no mention of use of duplicate review/data checks for abstraction	Yes
3. Was a comprehensive literature search performed?	Yes, search terms provided, but not exact search strategy	Yes, but only report search strategy for MEDLINE (table two)	Yes, but only report search strategy for MEDLINE (table one)	Yes, search terms provided -- Appendix B, Medline only	Yes (Identification of papers, Appendix one)
4. Was the status of publication (i.e., grey literature) used as an inclusion criterion?	No	No	No	No	Unclear
5. Was a list of studies (included and excluded) provided?	No, only included studies	No, only included studies	No, only included studies	Yes (Appendix A)	No, only included studies
6. Were characteristics of included studies provided?	Yes	Yes (tables four-ten)	Yes	Yes (Table two, Appendix C)	Yes (Tables two, five, seven)
7. Was the scientific quality of the included studies assessed and documented?	Yes (methods, results)	Yes (methods)	Yes (methods, table five)	Yes (Appendix D)	Yes (Tables one, four, six)
8. Was the scientific quality of the included studies used appropriately in formulating conclusions?	Yes	Yes	Yes	Yes	Yes
9. Were the methods used to combine the findings of studies appropriate?	Yes -- qualitative review appropriate	Yes -- qualitative review appropriate -- suggest stating explicitly	Yes -- qualitative review appropriate	Yes, qualitative review appropriate	Yes, qualitative review appropriate
10. Was the likelihood of publication bias assessed?	No	No	No	No	No
11. Were potential conflicts of interest included?	Yes	Yes (Acknowledge-ments)	Yes	Yes (Competing interests)	Yes

This table outlines the methodological quality assessment of each of the systematic reviews using the Assessment of Multiple Systematic Reviews tool (AMSTAR) [43].

To ensure transparency, the CSEP convened an independent expert panel to objectively assess the evidence presented by the review authors at an international Consensus Conference held in January 2009 in Kananaskis, Alberta. The panel was constituted to be as objective as possible with regard to the presentations of evidence. Therefore, three members including the chair (a majority), were from outside the physical activity field and had expertise from associated relevant disciplines (Public Health, Clinical Epidemiology, Medicine), and two members had subject-specific knowledge in physical activity and health; none of the expert panel worked on any aspect of the project to-date.

Prior to the Consensus Conference, panel members received specific guidance about their role from the Guidelines Chair (MST), copies of background material, and draft copies of the systematic reviews from each of the authors. At the Consensus Conference, the authors of each of the systematic reviews presented their papers to all delegates in a 30 minute presentation, followed by 30 minutes of general discussion by all delegates, and 30 minutes of protected discussion by the expert panel. Panel members clarified any areas about the evidence directly with authors. Throughout the Consensus Conference, panel members met in a separate room to discuss the evidence and develop recommendations. Using data from the systematic reviews, panel members carefully considered the strengths and weaknesses of the body of evidence from content validity and research design perspectives, including the risk of bias in study execution. The panel achieved unanimous consensus on the level of evidence informing the physical activity guidelines and developed a separate document outlining key recommendations, interpretation of the evidence and justification of each recommendation. This "consensus paper" is located in this collection of papers.

The guidelines will be circulated to experts and key stakeholders who were not involved in the guideline development process for feedback. Details regarding the process of stakeholder feedback and external review are still being determined. It is proposed to the Public Health Agency of Canada that the guidelines be reviewed and updated, as necessary, every 3-5 years on a rotating basis, or if clinically important research results necessitate change, whichever occurs first. This strategy would ensure that the physical activity guideline evidence for at least one of the population groups is reviewed and updated either annually or biannually. Further, the systematic review authors and guidelines chair (MST) will monitor the physical activity guidelines literature for changes in the field. As active investigators in exercise science, we are confident this strategy will identify clinically important trends and this approach is consistent with recent evidence on updating systematic reviews [45].

The *Clarity of Presentation *domain (4 items) addresses the language, formatting and structure of the guidelines, and barriers and facilitators of guideline implementation. Recommendations from the expert panel are clearly highlighted in the collection of papers here and are summarized in the consensus paper. For each recommendation, we sought to provide specific recommendations for each population, the rationale for each recommendation, and qualifying statements (as necessary). Because these guidelines were focused on physical activity, we did not present alternative strategies, such as dietary interventions. However we did provide suggestions regarding different types of physical activity for consideration. Specific head-to-head comparisons of different types of physical activity were beyond the scope of the systematic reviews and guidelines. To better understand the behavioural considerations of implementing physical activity guidelines, we included 2 systematic reviews in this collection of papers examining modifiable mediators of physical activity change and message framing. These reviews apply across the spectrum of the guideline populations, and provide evidence regarding important considerations for designing implementation studies of guideline recommendations.

The *Applicability *domain (3 items) includes advice for implementing recommendations, resource implications, and monitoring strategies. We anticipate that the Public Health Agency of Canada and the CSEP, in partnership with key stakeholders such as ParticipACTION, will develop and promote specific multimodal tools to help disseminate and implement the guidelines at the population level. For past versions of the guidelines, specific methods of dissemination included presentation at scientific meetings, open access journal supplements, posting guidelines on the internet, and mailings to key stakeholders. Canada's Physical Activity Guides and accompanying resources were made widely available free of charge through a country-wide toll free number and on-line - http://www.paguide.com. We expect to conduct similar, evidence-based dissemination and implementation strategies in the future. All materials are available in English and French, Canada's two official languages.

Resource implications of implementing the guidelines were beyond the scope of the systematic reviews and guidelines. Each guideline has specific information regarding physical activity targets for monitoring purposes. Monitoring physical activity is a controversial area in exercise science, as self-report measures overestimate physical activity compared to direct measures, such as that by actigraphy [46,47]. Development of medium and long-term evaluation strategies to assess the effect of these Guidelines in professional practice and health care outcomes are underway. Most recently, Statistics Canada initiated population-based measures of physical activity and health, including direct measures of activity [48]. We expect to assess the impact of the guidelines using data from these measures; however, guideline concordance assessment is beyond the scope of this document and this group.

The *Editorial Independence *domain (2 items) addresses potential biases in guideline recommendations due to funding source or guideline panel member conflicts of interest. All of the guidelines were funded by the Public Health Agency of Canada, whose views had no influence on the final recommendations presented in the guidelines. The Agency did provide guidance on areas of interest for the guidelines, but was not directly involved in evidence accumulation, synthesis, or interpretation. The second item in Editorial Independence addresses conflict of interest by guideline development members. Each development member included a conflict of interest statement in their review and none declared any conflicts of interest.

Please see Table 3 for a summary of AGREE II reporting items.

**Table 3:** AGREE II Reporting table for Canadian Physical Activity Guidelines

AGREE II Item	Reporting Location in Physical Activities Guidelines	Internal AGREE II score
**Domain 1. Scope and Purpose**		
1. The overall objective(s) of the guideline is (are) specifically described.	Process paper, table 1	7
2. The health question(s) covered by the guideline is (are) specifically described.	Process paper, table 1	7
3. The population (patients, public, etc.) to whom the guideline is meant to apply is specifically described.	Process paper, table 1	7
**Domain 2. Stakeholder Involvement**		
4. The guideline development group includes individuals from all the relevant professional groups.	Process paper, Stakeholder Involvement description	7
	Process paper, Rigour of development description	
	Process paper, Table 2	
5. The views and preferences of the target population (patients, public, etc.) have been sought.	Process paper, Stakeholder Involvement description	1
6. The target users of the guideline are clearly defined.	Consensus paper, Review section, paragraph 3	7
**Domain 3. Rigour of Development**		
7. Systematic methods were used to search for evidence.	Please see each of the systematic reviews for information on this item	7
8. The criteria for selecting the evidence are clearly described.	Please see each of the systematic reviews for information on this item	7
9. The strengths and limitations of the body of evidence are clearly described.	Please see each of the systematic reviews for tables outlining the risk of bias of individual studies	5
	Process paper, Rigour of Development description	
	Process paper, Table 3	
10. The methods for formulating the recommendations are clearly described.	Process paper, Rigour of Development description	7
	Consensus paper, Review section	
11. The health benefits, side effects and risks have been considered in formulating the recommendations.	Consensus paper, adverse effects section	7
12. There is an explicit link between the recommendations and the supporting evidence.	Consensus paper recommendations	1
13. The guideline has been externally reviewed by experts prior to its publication.	Process paper, Rigour of Development description	7
14. A procedure for updating the guideline is provided.	Process paper, Rigour of Development description	7
**Domain 4. Clarity of Presentation**		
15. The recommendations are specific and unambiguous.	Process paper, Clarity of Presentation description	7
	Consensus paper	
16. The different options for management of the condition or health issue are clearly presented.	Process paper, Clarity of Presentation description	7
17. Key recommendations are easily identifiable.	Consensus paper	7
18. The guideline describes facilitators and barriers to its application.	Process paper, Clarity of Presentation description	1
**Domain 5. Applicability**		
19. The guideline provides advice and/or tools on how the recommendations can be put into practice.	Process paper, Applicability description	7
20. The potential resource implications of applying the recommendations have been considered.	Process paper, Applicability description	1
21. The guideline presents monitoring and/or auditing criteria.	Process paper, Applicability description	7
**Domain 6. Editorial Independence**		
22. The views of the funding body have not influenced the content of the guideline.	Process paper, Editorial Independence description	7
23. Competing interests of guideline development group members have been recorded and addressed.	Process paper, Editorial Independence description	7
	Consensus paper, Competing interests	
	Systematic reviews	

This table outlines the different documents where readers will find information to complete quality assessment of the guidelines development process using the AGREE II instrument [19,20]. The Internal AGREE score represents the AGREE II item score assessed by one of the consultant methodologists (ACT). Description of reporting locations: Process paper, this document; Consensus paper, Kesäniemi et al., 2009 [42]; Systematic reviews, Children and Youth: Janssen et al., 2009 [30]; Adults: Warburton et al., 2009 [31]; Older Adults: Paterson et al., 2009 [32].

## Discussion

Research examining the relationship between physical activity and health has proliferated in recent years and the evidence informing physical activity guidelines has expanded significantly. Because the existing guidelines were based on evidence that was several years old, the CSEP and Public Health Agency of Canada began a project to review the existing guidelines and guides [18,28]. In this report, we described the process the CSEP PAMG Steering Committee followed to produce clear, evidence-based physical activity recommendations for school-aged children (5-17 years), adults (18-64 years) and older adults (≥65 years).

To our knowledge, this is the first effort to develop physical activity guidelines using internationally-recognized standards for guideline development [19,20,40]. We undertook a medically-recognized, formal and rigorous approach to the review of the exercise science evidence [41]. Using the AMSTAR tool, all of the SRs that were used to formulate the CPGs were rated as being moderate to high quality, overall. We graded evidence using a similar system to that of existing Canadian guidelines in this area [36,38], and used the methodology to distil and interpret the evidence to inform recommendations.

Feedback from participants and attendees following the 2009 International Consensus conference indicated the process used to develop CPGs for physical activity by the CSEP PAMG Steering Committee was rigorous, objective and thorough. Based on the presentations from the invited international physical activity experts at the 2009 Consensus Conference [49], we learned our process reported herein was similar in many respects to that of other physical activity guideline developers (e.g., Unites States, United Kingdom, Australia, World Health Organization). Indeed, the other guideline development groups lauded our efforts and suggested that CSEP had raised the standard for physical activity guideline development internationally. To our knowledge, this is the first effort to use a methodologically rigorous formal guideline development framework and systematic reviews to inform consensus recommendations in physical activity.

Other guideline development agencies support the CSEP guideline development approach [50]. Three main strengths of the PAMG Steering Committee approach included focusing on specific physical activity questions important to Canadians in different age groups for the systematic reviews, use of a common metric to assess evidence quality, and initiating collaboration among international organizations [50]. Building on previous experiences, CSEP and its stakeholders are actively developing ways to help guideline consumers understand and implement the guidance statements.

### Strengths, weaknesses, limitations

Strengths of our approach include use of a multidisciplinary team to gather and synthesize the evidence, involvement of a range of stakeholders to interpret the evidence, independence of the expert panel, and development of proactive plans for guideline dissemination, implementation, and evaluation. Our multidisciplinary team included exercise science content experts and methodological experts. Our consensus conference assembled international experts in exercise science, and representatives of end users of the guidelines (e.g., Aboriginal populations). Building on existing relationships from our previous guides, we are planning rigorous strategies to disseminate, implement, and evaluate the guidelines.

Our approach also has limitations. Based on data from the systematic reviews, the state of the evidence limited our ability to make more specific recommendations or provide the level of detail desired for justifying the recommendations. Although the available evidence has significantly increased since publication of the first Canadian physical activity guidelines, more research is required on structured, population-based samples examining specific health outcomes for all age groups, with consideration of study design, subject participation levels and specificity. Studies of the mediators of physical activity and effective messaging of physical activity guidelines, both critically important areas, are relatively new and there is clearly scope for further investigation. According to the AMSTAR assessments, the systematic reviews had some limitations. None of the reviews included unpublished material or formally assessed for publication bias and only one provided a list of excluded studies. Systematic reviews used in future updates of the guidelines will attempt to surmount these limitations.

Reports of conflicts of interest and influence of guideline sponsorship on recommendations are areas potentially influenced by drug and device interventions; the generalizability of these concepts to physical activity guidelines is a topic requiring further study.

### Next Steps

The next phase of the project (Phase 4) is planned to unfold during the latter part of 2009 and 2010 and will consist of the final writing of the CPGs by a CPG Writing Group in consultation with the Canadian Vascular Coalition, C-CHANGE and the Canadian Prevention Task Force on Preventive Health Care. The CSEP PAMG Steering Committee will seek formal endorsement from key leaders and organizations followed by implementation in a wide range of health care settings and the general public. Depending on resources (which shall dictate scope), a dissemination plan and evaluation strategy shall be developed to assess the initial impact of the CPGs for asymptomatic populations.

### Development of physical activity guidelines for symptomatic populations

Concurrent to the process outlined above, the CSEP has undertaken a related project to examine the development of CPGs for symptomatic populations (those with chronic diseases or conditions) through a parallel project funded by Public Health Agency of Canada entitled "Development of Evidence-Based Clinical Physical Activity Prescription Guidelines for Chronic Disease" http://www.csep.ca using the same rigorous approach. Canadian CPGs for physical activity for both asymptomatic and symptomatic population segments should contribute to enhancing population health in this key area of public health.

## Conclusion

Over the past three years the CSEP and the Public Health Agency of Canada have followed a rigorous process to examine the evidence informing potential revisions to existing physical activity guidelines for Canadians. Believed to be the first physical activity guideline development process in the world to be guided and assessed by AGREE (and certainly the first to use AGREE II) and AMSTAR instruments, the PAMG Steering Committee also intends to see this process through to the development of CPGs, also believed to be a first. The CSEP PAMG Steering Committee recommends that a similarly rigorous approach be considered by all jurisdictions developing or revising physical activity guidelines. Going forward, and based on international efforts to coordinate chronic obstructive pulmonary disease [50], areas where CSEP could undertake a leadership role include the development of a global physical activity evidence database, initiation of collaborative evidence reviews among organizations, and examination of collaborative models for funding guideline development and implementation.

## Appendix A

**Steering Committee on Advancing the Future of Physical Activity Measurement and Guidelines (December 2006, Ottawa, Canada):** 

**Panel Member**	**Role/Affiliation**
Mark Tremblay	University of Saskatchewan, Think Tank Chair
Dale Esliger	University of Saskatchewan, Project Manager
Larry Brawley	University of Saskatchewan
Cora Craig	Canadian Fitness and Lifestyle Research Institute
Peter Katzmarzyk	Queen's University
Lori Zehr	Canadian Society for Exercise Physiology, Health and Fitness Program Chair
William Hearst	Healthy Living Unit, Public Health Agency of Canada
Randy Adams	Physical Activity Unit, First Nations and Inuit Health Branch, Health Canada
Mary Duggan	Canadian Society for Exercise Physiology, Manager

Appendix B

Members of the Canadian Vascular Coalition

Canadian Association of Cardiac Rehabilitation

Canadian Diabetes Association

Canadian Council for Tobacco Control

Canadian Hypertension Education Program

Canadian Society for Exercise Physiology

Canadian Working Group on Hypercholesterolemia and other Dyslipidemias

Obesity Canada

Appendix C

**Conference Delegates:** 

**Name**	**Institution**	**Role**
Mark Tremblay, Ph.D.	Children's Hospital of Eastern Ontario,	Chair
	Ottawa, Canada	
Antero Kesaniemi, Chair M.D., Ph.D.	Department of Internal Medicine, University of Oulu, Finland	Expert panel
Bruce Reeder, M.D. MHSc, FRCPC	Department of Community Health and Epidemiology, College of Medicine, University of Saskatchewan, Saskatoon, Saskatchewan, Canada	Expert Panel
Chris Riddoch, Ph.D.	School for Health, Bath University, Bath, U.K.	Expert Panel
Thorkild Sorensen, Dr.Med.Sci.	Professor of Clinical Epidemiology and Institute Director, Institute of Preventive Medicine, Copenhagen University Hospital, Copenhagen, Denmark	Expert Panel
Steven Blair, Ph.D.	Department of Exercise Science, University of South Carolina, Columbia, South Carolina U.S.A.	Expert Panel
Ian Janssen, Ph.D. Speaker	School of Kinesiology and Health Studies, Queen's University, Kingston, Canada	Systematic review author, gap areas (youth, 15-19 years)
Darren Warburton, Ph.D.	School of Human Kinetics, University of British Columbia, Vancouver, B.C., Canada	Systematic review author
Donald Paterson, Ph.D.	School of Kinesiology, The University of Western Ontario, London, Ontario, Canada	Systematic review author
Amy Latimer, Ph.D.	School of Kinesiology and Health Studies Queen's University, Kingston, Canada	Systematic review author
Ryan Rhodes, Ph.D.	Behavioural Medicine Laboratory, Faculty of Education, University of Victoria, Victoria, B.C. Canada	Systematic review author
Vanessa Candeias	Department of Chronic Diseases and Health Promotion, World Health Organization, Geneva, Switzerland	World Health Organization
Stuart Biddle, Ph.D.	School of Sport and Exercise Sciences, Loughborough University, Leicestershire, U.K.	International representative
Richard Troiano, Ph.D.	U.S. Dept of Human Health Services, Office of Public Health and Science, Washington, D.C., U.S.A.	International representative
Trevor Shilton	National Heart Foundation of Australia Perth, Australia	International representative
Brian Timmons, Ph.D.	Department of Pediatrics, Chedoke-McMaster, Hamilton, Ontario, Canada	Speaker, gap areas Preschool children
Michelle Mottola, Ph.D.	School of Kinesiology, The University of Western Ontario, London, Ontario, Canada	Speaker, gap areas - Pregnant women
Kathleen Martin Ginis	Department of Kinesiology, McMaster University, Hamilton, Ontario, Canada	Speaker, gap areas - Disability
Peter Katzmarzyk, Ph.D.	Pennington Biomedical Research Center, Baton Rouge, Louisianna, U.S.A.	Speaker, gap - Aboriginal
William Haskell, Ph.D.	Stanford Prevention Research Center, Stanford University School of Medicine, Stanford, California, U.S.A.	International representative
Roy Shephard, M.D., Ph.D., D.P.E.	Professor Emeritus, Faculty of Physical Education and Health, University of Toronto Toronto, Ontario, Canada	Content expert
I-Min Lee, M.D., Sc.D.	Department of Epidemiology, Harvard School of Public Health, Boston, Massachusetts, U.S.A.	Content expert
Norm Gledhill, Ph.D.	School of Kinesiology and Health Science, York University, Toronto Ontario, Canada	Content expert
James Stone, M.D., Ph.D., FRCPC	Department of Cardiology, Foothills Hospital, Calgary, Alberta, Canada	Content expert
Russell Pate, Ph.D.	Arnold School of Public Health, University of South Carolina, Columbia, South Carolina, U.S.A.	Content expert
Rod Dishman, Ph.D.	College of Education, University of Georgia, Athens, Georgia, U.S.A.	Content expert
Van Hubbard, M.D., Ph.D.	National Institutes of Heath, Division of Nutrition Research Coordination, Bethesda, Maryland, U.S.A.	Content expert
Michelle Kho, PT, MSc, Ph.D.(c)	McMaster University, Hamilton, Ontario, Canada	Methodological consultant, clinical practice guidelines (AGREE)
Andrea Tricco, Ph.D.(c)(telephone)	University of Ottawa, Ottawa, Ontario, Canada	Methodological consultant, systematic reviews
Christine Cameron Ph.D.	Canadian Fitness and Lifestyle Research Institute, Ottawa, Ontario, Canada	Steering committee
Lawrence Brawley, Ph.D.	College of Kinesiology, University of Saskatchewan, Saskatoon, Canada	Steering committee
Lori Zehr, MSc.	Centre for Sport & Exercise Education, Camosun College, Victoria, B.C., Canada	Steering committee
Brian MacIntosh, Ph.D.	Faculty of Kinesiology, University of Calgary, Calgary, Alberta, Canada	Steering committee
Angelo Belcastro, Ph.D.	Faculty of Kinesiology, University of New Brunswick, Fredericton, N.B., Canada	Steering committee
Mary Duggan	Manager, Canadian Society for Exercise Physiology, Ottawa, Canada	Steering committee
Ashlee McGuire, Ph.D.(c)	School of Kinesiology and Health Studies, Queen's University, Kingston, Ontario, Canada	Steering committee
Sarah Charlesworth Ph.D.	School of Human Kinetics, University of British Columbia, Vancouver, B.C., Canada	Steering committee
Kelly Murumets MSW, MBA	President and CEO, ParticipACTION, Toronto, Ontario, Canada	Delegate
Art Salmon, Ed.D.	Team Leader: Research, Ontario Ministry of Health Promotion, Toronto, Ontario, Canada	Delegate
Isabel Romero	Director, Healthy Communities Division, Public Health Agency of Canada, Ottawa, Canada	Delegate
Randy Adams, MBA	Research Manager, Public Health Agency of Canada, Ottawa, Canada	Delegate
Halina Cyr	Director, Chronic Disease and Injury Prevention, Division, First Nations and Inuit Health Branch, Health Canada, Ottawa, Canada	Delegate
Julie Pinard	Physical Activity Specialist, First Nations and Inuit Health Branch, Health Canada, Ottawa, Canada	Delegate

## Conflicts of interests

MD is employed by the Canadian Society for Exercise Physiology. MK and AT received consulting fees from the Canadian Society for Exercise Physiology.

## Authors' contributions

MST conceived of and led the development, preparation and writing of the manuscript. MEK and ACT provided expert input and direction on the process assessment elements of the paper and also performed the assessment scoring for the AMSTAR and AGREE instruments. MD provided assistance with the writing, editing and organization of all process elements described and evaluated in the manuscript. All authors read and approved the final manuscript.
